# Evaluating the risk of atrial fibrillation in patients with chronic recurrent pericarditis prescribed colchicine: Observations using TriNetX global federated research network

**DOI:** 10.1007/s00228-025-03925-4

**Published:** 2025-12-18

**Authors:** Mert Kaşkal, Tommaso Bucci, Muath Alobaida, Steven Ho Man Lam, Michele Rossi, Enrico Tartaglia, Amir Askarinejad, Andrea Galeazzo Rigutini, Gregory Y. H. Lip, Rebecca A. B. Burton

**Affiliations:** 1 Liverpool Centre for Cardiovascular Science at University of Liverpool, Liverpool John Moores University and Liverpool and Heart and Chest Hospital, Liverpool, UK; 2 Department of Medical Pharmacology, School of Medicine, Marmara University, Istanbul, Turkey; 3 Department of Clinical Internal, Anesthesiologic and Cardiovascular Sciences, Sapienza University of Rome, Rome, Italy; 4 Department of Basic Science, Prince Sultan Bin Abdulaziz College for Emergency Medical Services, King Saud University, Riyadh, Saudi Arabia; 5 Department of Life, Health & Environmental Sciences, University of L’Aquila, L’Aquila, Italy; 6 Internal Medicine and Nephrology Division, ASL1 Avezzano-Sulmona-L’Aquila, San Salvatore Hospital, L’Aquila, Italy; 7 Cardiology Division, Department of Biomedical, Metabolic and Neural Sciences, Italy University of Modena and Reggio Emilia, Policlinico di Modena, Modena, Italy; 8 Rajaie Cardiovascular Medical and Research Institute, Iran University of Medical Sciences, Tehran, Iran; 9 Internal, Vascular and Emergency Medicine – Stroke Unit, University of Perugia, Perugia, Italy; 10 Department of Clinical Medicine, Aalborg University, Aalborg, Denmark; 11 Department of Cardiology, Lipidology and Internal Medicine with Intensive Coronary Care Unit, Medical University of Bialystok, Bialystok, Poland; 12 Department of Pharmacology, University of Oxford, Oxford, UK; 13 Institute of Systems, Molecular and Integrative Biology, Department of Pharmacology and Therapeutics, University of Liverpool, Liverpool, UK

**Keywords:** Chronic pericarditis, Colchicine, Atrial fibrillation

## Abstract

**Background:**

Chronic pericarditis is associated with significant cardiovascular morbidity, including atrial fibrillation (AF), heart failure (HF), and stroke. Colchicine is widely used in pericarditis management for its anti-inflammatory effects, but its impact on arrhythmias and other cardiovascular outcomes remains uncertain.

**Methods:**

We conducted a retrospective observational cohort study using the TriNetX global federated research network to assess the association between colchicine use and cardiovascular outcomes in in-patients with chronic pericarditis. Patients diagnosed with chronic adhesive and constrictive pericarditis with or without colchicine use between 2010 and 2024 were included. Propensity score matching (PSM) (1:1) was used to balance baseline characteristics. The primary outcome was 1-year incidence of AF. Secondary outcomes included all-cause mortality, ischemic stroke, acute myocardial infarction (AMI), acute HF, cardiac arrest, ventricular arrhythmias, and a composite cardiovascular outcome.

**Results:**

Of 8,120 patients hospitalized with chronic pericarditis, 1,064 received colchicine. 1,061 patients were matched in each group after PSM. The 1-year incidence of AF was similar between colchicine users and non-users (HR: 0.90, 95% CI: 0.76–1.06) after PSM. Colchicine use was associated with significantly lower risk of all-cause mortality (HR: 0.67, 95% CI: 0.54–0.84) and ischemic stroke (HR: 0.45, 95% CI: 0.29–0.67) after PSM. No significant differences were observed for AMI, cardiac arrest, HF, or ventricular arrhythmias.

**Conclusion:**

In this real-world cohort of patients with chronic pericarditis, colchicine did not increase the risk of AF. Colchicine was linked to significantly lower risks of all-cause mortality and ischemic stroke in hospitalized chronic pericarditis patients, suggesting potential systemic cardiovascular benefits.

**Supplementary Information:**

The online version contains supplementary material available at 10.1007/s00228-025-03925-4.

## Introduction

Chronic pericarditis is an inflammatory condition that can lead to substantial cardiovascular morbidity, including atrial fibrillation (AF), heart failure (HF), and thromboembolic complications [1]. Colchicine, a well-established anti-inflammatory agent, has been widely used in the management of pericarditis due to its ability to reduce recurrences and systemic inflammation [2]. A dose of 1 mg/day is generally used for a minimum of 6 months for the treatment and control of chronic recurrent pericarditis [3]. Colchicine has been shown to be a microtubule disrupting agent [4, 5]. Through inhibition of microtubule polymerization and suppression of pro-inflammatory cytokines such as interleukin-1β and interleukin-6, colchicine is believed to have protective cardiovascular effects [6].

Colchicine has been studied for its effects on cardiac arrhythmias, particularly AF. While some studies suggest that colchicine may reduce the incidence of AF in specific contexts, there is also evidence indicating potential pro-arrhythmic effects, especially concerning ventricular arrhythmias [7]. Despite growing interest in colchicine’s cardiovascular applications, its role in modifying the risk of arrhythmias and ischemic events among patients with chronic pericarditis remains unclear. Furthermore, limited real-world evidence is available to evaluate its impact on outcomes such as AF, stroke, acute myocardial infarction (AMI), HF and cardiac arrest in this specific population.

In this retrospective observational cohort study using the TriNetX global federated research network, we aimed to assess whether colchicine use is associated with a reduced risk of AF and other major cardiovascular events in patients with chronic pericarditis. Our primary objective was to examine the 1-year risk of AF among colchicine users versus non-users, while secondary objectives included the evaluation of all-cause mortality, ischemic stroke, AMI, acute HF, cardiac arrest, and composite cardiovascular outcomes.

## Methods

### Study design

This study was designed as a retrospective observational analysis utilizing data from TriNetX, a global federated research network that uses electronic medical records from a wide range of healthcare institutions. These include academic medical centers, specialty clinics, and community hospitals, collectively representing health data from approximately 300 million individuals worldwide but the majority of its data comes from healthcare organizations in the United States. The TriNetX network includes some data from Europe, Latin America, and Asia, the majority of patients are from U.S.-based institutions, resulting in a predominantly white population (approximately 65–75%, depending on the cohort and condition) as the source of their health database. Additional information regarding the platform can be found at https://trinetx.com.

The available dataset within TriNetX includes information such as demographic characteristics, diagnostic codes (classified according to ICD-9-CM and ICD-10-CM standards), and prescription data, which are coded using Veteran Affairs (VA) medication codes. TriNetX adheres to the standards set by the Health Insurance Portability and Accountability Act (HIPAA) and complies with relevant U.S. federal regulations that protect the privacy and security of health information. All data used for analysis are anonymized in accordance with the HIPAA Privacy Rule. As TriNetX operates on a federated network model, analyses conducted using its platform do not require institutional ethical approval, as no identifiable patient-level data can be accessed.

### Study cohort

Data searches were conducted using the TriNetX platform. We compared the baseline characteristics of colchicine users and non-users among patients diagnosed with chronic adhesive pericarditis (ICD-10-CM: I31.0) or chronic constrictive pericarditis (ICD-10-CM: I31.1) between January 1, 2010, and January 1, 2024.

For the colchicine user group, the index event was defined as a hospital inpatient or observation care encounter associated with a diagnosis of chronic adhesive or constrictive pericarditis (ICD-10-CM: I31.0 or I31.1), followed by the initiation of colchicine therapy. For the non-user group, the index event was similarly defined as a hospital inpatient or observation care encounter with a diagnosis of chronic adhesive or constrictive pericarditis, but without colchicine use. The decision to focus on hospitalized patients was intended to reduce heterogeneity and improve diagnostic accuracy. Individuals with a previous diagnosis of AF were not included in the analysis. Additional details regarding the inclusion and exclusion criteria, along with the specific ICD-10 codes, are provided in Supplementary Table 1.

### Outcomes

Outcomes were assessed over a 1-year follow-up period, defined as days 5 to 370 following the index event. Outcome assessment began five days after hospitalization to allow for the initiation of colchicine therapy during the early inpatient period. The primary outcome was the comparison of 1-year risk of AF and flutter (ICD-10-CM: I48) in patients with chronic pericarditis with and without the use of colchicine. The secondary outcomes for the study are 1-year all-cause mortality, composite events (AMI (ICD-10-CM: I21), mortality, acute systolic HF (ICD-10-CM: I50.21), acute diastolic HF (ICD-10-CM: I50.31), cardiac arrest (ICD-10-CM: I46), ventricular fibrillation and flutter (ICD-10-CM: I49), AF and flutter (ICD-10-CM: I48), ischemic stroke (ICD-10-CM: I63) ) we also look at the 1-year risk separately for AMI (ICD-10-CM: I21), acute HF(ICD-10-CM: I50.21 or I50.31), cardiac arrest (ICD-10-CM: I46), ventricular fibrillation and flutter (ICD-10-CM: I49), ischemic stroke (ICD-10-CM: I63) as secondary outcomes.

### Statistical analysis

Baseline characteristics between colchicine users and non-users were balanced using logistic regression and propensity score matching (PSM) at a 1:1 ratio. Matching was performed based on age, sex, race, the diagnosis for arterial hypertension, hyperlipidemia, diabetes mellitus, chronic kidney failure obesity, anemias, ischemic heart disease and for the drugs such as antianginals, antiarrhythmics (Class I and III), ACE (angiotensin-converting-enzyme) inhibitors, ARBs (Angiotensin II receptor blockers), beta blockers, diuretics, statins, aspirin, antithrombotics (heparin, vitamin k antagonists, direct factor Xa inhibitors, direct thrombin inhibitors), long-term use of NSAIDs (non-steroid anti-inflammatory drugs).

The balance of demographic and clinical variables between groups was evaluated using absolute standardized differences (ASD), (Supplementary Fig. 1). These variables were selected based on their potential association with cardiovascular risk. Following matching, Cox proportional hazards models were used to calculate hazard ratios (HRs) and 95% confidence intervals (CIs) for the predefined outcomes, comparing colchicine users with non-users. Kaplan–Meier survival curves were constructed to illustrate differences in event-free survival between groups for both primary and secondary outcomes. Log-rank tests were used to assess between-group differences in the probability of experiencing the outcome over time.

All analyses were executed within the TriNetX platform, which utilises both R and Python for data analysis. The R Survival library v3.2-3 was used for survival analyses, while propensity risk scores were estimated using logistic regression, implemented via the scikit-learn package in Python version 3.7. All tests were two-tailed and statistical significance was defined as *p*-values < 0.05, indicating assuming a Type I error of less than 5% if the null hypothesis is true.

## Results

In this retrospective propensity score matched analysis of colchicine effect on AF in patients with chronic pericarditis, we have identified a total of 8,120 patients with chronic pericarditis. Among these patients 7,056 were not prescribed colchicine (mean age 58.8, SD 19.8; 42.1% female), 1,064 were given colchicine (mean age 57.0, SD 17.2, 44.6% female).

The median follow-up in non-colchicine group before propensity score matching (PSM) was 370 days (range 300–440 days) and in colchicine group was 352 days (range 280–424 days). Before PSM, notable imbalances were observed between colchicine users and non-users among patients with pericarditis (Table 1).

**Table 1:** Baseline characteristics comparison between Colchicine users and non-user before and after propensity score matching

Baseline characteristics	Before propensity score matching			After propensity score matching		
	Pericarditis with colchicine use (*n*=1,064)	Pericarditis with no colchicine use (*n*=7,056)	ASD	Pericarditis with colchicine use (*n*=1,061)	Pericarditis with no colchicine use (*n*=1,061)	ASD
Age, years (±SD)	57 ± 17.2	58.8 ± 19.8	0.096	57.1 ± 17.2	58.5 ± 19.8	0.081
Female, n (%)	461 (44.6)	2,946 (42.1)	0.032	462 (44.7)	455 (44.1)	0.013
White, n (%)	735 (69.1)	5,058 (71.7)	0.057	735 (69.3)	755 (71.2)	0.041
Arterial hypertension, n (%)	744 (69.9)	4,922 (69.7)	0.003	743 (70.0)	750 (70.7)	0.014
Hyperlipidemia, n (%)	504 (47.4)	3,437 (48.7)	0.029	504 (47.5)	523 (49.3)	0.035
Diabetes mellitus, n (%)	343 (36.4)	2,023 (28.7)	0.166	387 (36.4)	387 (36.4)	0.000
Chronic kidney failure, n (%)	291 (27.3)	2,265 (32.1)	0.104	291 (27.4)	298 (28.1)	0.014
Obesity, n (%)	388 (36.5)	2,023 (28.7)	0.166	387 (36.4)	387 (36.4)	0.000
Anemias, n (%)	636 (59.8)	3,549 (50.2)	0.191	743 (70.0)	750 (70.7)	0.014
Ischemic heart disease, n (%)	498 (46.8)	3,737 (52.9)	0.123	498 (46.9)	517 (48.7)	0.035
Antiarrythmics, n (%)	837 (78.6)	4,694 (66.5)	0.274	834 (78.6)	836 (78.8)	0.004
ACE inhibitors, n (%)	305 (28.8)	2,169 (30.7)	0.045	305 (28.7)	308 (29.0)	0.006
ARBs, n (%)	226 (21.2)	1,452 (20.6)	0.016	226 (21.3)	238 (22.4)	0.027
Beta blockers, n (%)	714 (67.1)	4,483 (63.5)	0.075	714 (67.3)	741 (69.8)	0.054
Diuretics, n (%)	746 (70.1)	4,674 (66.2)	0.083	745 (70.2)	771 (72.7)	0.054
Antithrombotics, n (%)	953 (89.6)	5,268 (74.7)	0.396	950 (89.5)	964 (90.8)	0.044
Aspirin, n (%)	635 (59.7)	4,004 (56.7)	0.059	633 (59.7)	650 (61.3)	0.032
Long-term use of NSAIDs, n (%)	61 (5.7)	211 (2.9)	0.134	58 (5.7)	58 (5.7)	0.000
Antianginals, n (%)	375 (35.2)	2,446 (34.7)	0.012	375 (35.3)	409 (38.5)	0.066
Statins	521 (48.9)	3,526 (49.9)	0.020	520 (49.0)	539 (50.8)	0.035

Following PSM, the baseline characteristics between the colchicine and non-colchicine groups were well balanced, with all ASD falling below the commonly accepted threshold of 0.1 (Table 1). The median follow-up in non-colchicine group after PSM was 312 days (range 255–369 days) and in colchicine group was 298 days (range 235–361 days).

Before PSM, colchicine users (*n* = 1,064) exhibited lower 1 - year incidence rates for several adverse cardiovascular outcomes compared to non-users (*n* = 7,056). Following PSM, where both groups included 1,061 patients, most incidence rates remained comparable or showed modest differences. The incidence of AF was 26.9% in colchicine users versus 29.0% in non-users before PSM. After PSM, 1 - year incidence of AF was 26.4% in colchicine users and 27.3% in non-users. The incidence of composite outcome was 48.4% in colchicine users, compared to 52.7% in non-users before PSM. After PSM the incidence was 48.2% in the colchicine group and 51.9% in the non-colchicine group (Table 2).

**Table 2:** Comparison of the 1-year risk of primary and secondary outcomes between Colchicine users and non-users in patients with chronic pericarditis

	Before Propensity Score Matching				After Propensity Score Matching			
	Colchicine users (*n*=1,064)	Non-colchicine users (*n*=7,056)	HR (95%CI)	*p*-value	Colchicine users (*n*=1,061)	Non-colchicine users (*n*=1,061)	HR (95%CI)	*p*-value
Number of AF (1-year) (n,%)	286 (26.9)	2,047 (29.0)	0.89 (0.79-1.02)	0.23	281 (26.4)	290 (27.3)	0.90 (0.76-1.06)	0.07
Number of composite events (1-year) (n,%)	515 (48.4)	3,720 (52.7)	0.82 (0.65-1.04)	0.38	512 (48.2)	551 (51.9)	0.86 (0.63-1.16)	0.20
Number of deaths (1 year) (n,%)	122 (11.5)	1,221 (17.3)	0.68 (0.57-0.82)	0.01	121 (11.4)	187 (17.6)	0.67 (0.54-0.84)	<0.0001
Number of cardiac arrests (1 year) (n,%)	22 (2.1)	233 (3.3)	0.58 (0.38-0.89)	0.04	22 (2.1)	18 (1.7)	0.64 (0.38-1.09)	0.63
Number of ischemic strokes (1-year) (n,%)	28 (2.6)	445 (6.3)	0.45 (0.31-0.63)	<0.0001	25 (2.4)	74 (7.0)	0.45 (0.29-0.67)	<0.0001
Number of AMI (1-year) (n,%)	65 (6.1)	586 (8.3)	0.75 (0.59-0.96)	0.04	61 (6.7)	79 (7.4)	0.83 (0.61-1.15)	0.41
Number of ventricular arrythmias (1-year) (n,%)	12 (1.1)	106 (1.5)	0.62 (0.34-1.16)	0.27	11 (1.0)	15 (1.4)	0.65 (0.30-1.41)	0.56
Number of acute HF (1-year) (n,%)	73 (6.9)	473 (6.7)	1.03 (0.82-1.30)	0.53	64 (6.0)	84 (7.9)	0.86 (0.64-1.13)	0.13

Notably, the 1-year incidence of mortality rate was 11.5% in the colchicine group and 17.3% in the non-colchicine group before PSM. After PSM 1-year mortality incidence was 11.4% in colchicine users compared to 17.6% in non-users. Before PSM, cardiac arrest incidence was 2.1% in colchicine users and 3.3% compared to non-users. After PSM, 1-year incidence of cardiac arrest was 2.1% in colchicine users and 1.7% in non-users. The 1-year incidence of ischemic stroke was 2.6% among colchicine users compared to 6.3% in non-users before PSM. After matching ischemic stroke incidence was 2.4% in colchicine group and 7.0% in non-colchicine group. Additionally, among colchicine users, the incidence of AMI was 6.1% compared to 8.3% in the non-colchicine group, and the incidence of ventricular arrhythmias was 1.1% versus 1.5% before PSM. The incidence of AMI was 6.7% and 7.4%, colchicine user’s vs. non-users, while the incidence of ventricular arrhythmias were 1.0% and 1.4% respectively after PSM. The incidence of acute HF was similar between groups, with 6.9% in colchicine users and 6.7% in non-users. After PSM the incidence of acute HF was 6.0% in colchicine users versus 7.9% in non-users (Table 2).

Before PSM, colchicine use in patients with chronic pericarditis was associated with a numerically low risk of AF during 1 - year after colchicine use (Hazard Ratio (HR): 0.89, 95% CI: 0.79–1.02; *p* = 0.27) but this was not statistically significant. For the secondary outcomes, colchicine use is associated with a significantly lower risk of all-cause death (HR: 0.68, 95% CI: 0.57–0.82; *p* = 0.01), cardiac arrest (HR: 0.58, 95% CI: 0.38–0.89; *p* = 0.03), ischemic stroke (HR: 0.45, 95% CI: 0.31–0.63; *p* < 0.0001), and AMI (HR: 0.75, 95% CI: 0.59–0.96; *p* = 0.04).

**Fig. 1:**
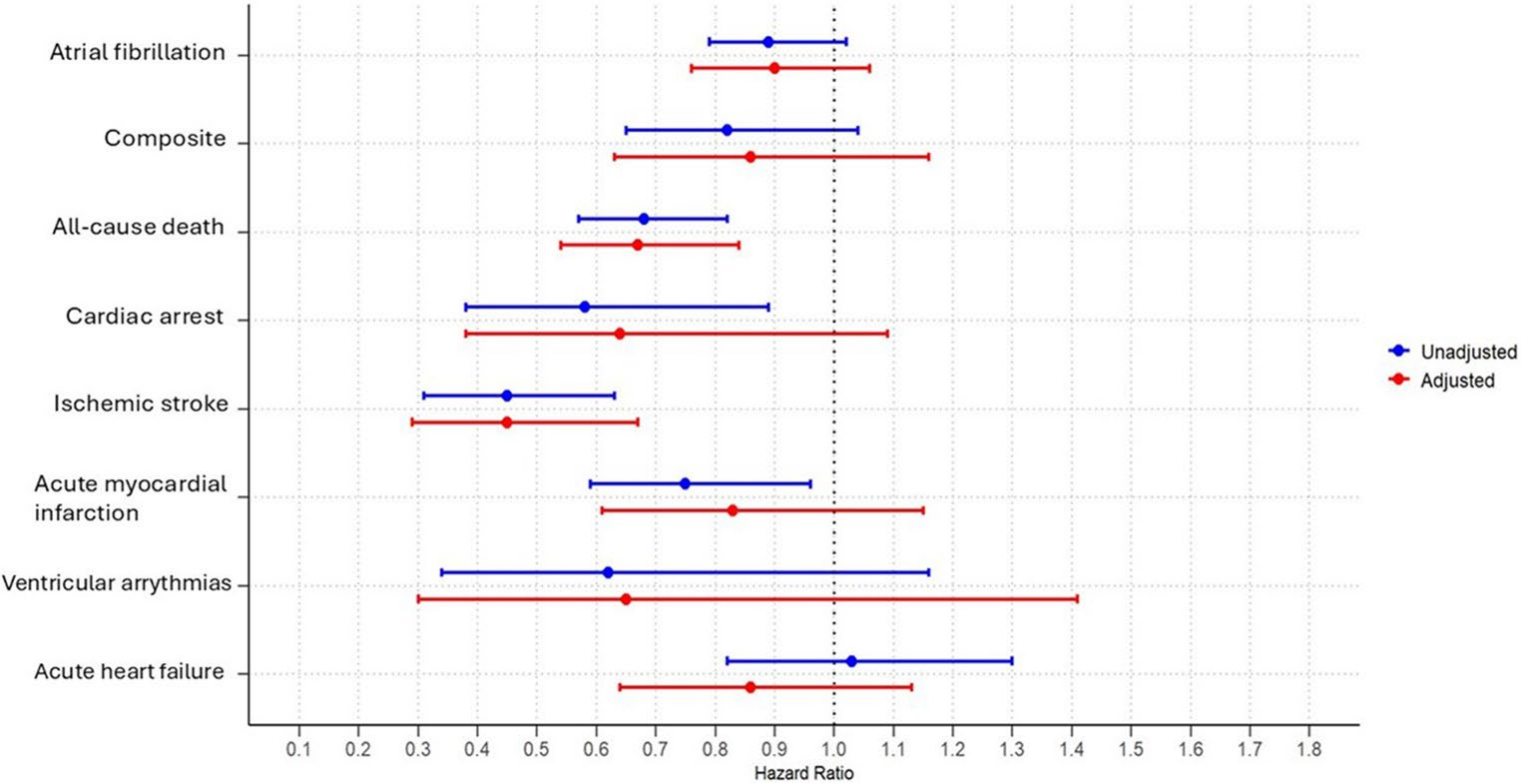
Forest plot analysis of hazard ratios and 95% confidence intervals for primary and secondary outcomes in colchicine users versus non-users with chronic pericarditis

The composite outcome for the cardiac events and ventricular arrythmias was numerically lower amongst colchicine users (HR:0.82, 95%CI: 0.65–1.04; *p* = 0.36 and HR:0.62, 95%CI: 0.34–1.16; *p* = 0.27; respectively), but this was not statistically significant. Colchicine use did not affect the risk of acute HF (HR: 1.03, 95% CI: 0.82–1.30; *p* = 0.54), (Table 2; Fig. 1).

After PSM, patients with colchicine had a lower number of AF events compared to non-colchicine users (HR: 0.90, 95%CI: 0.76–1.06; *p* = 0.07), but this was not statistically significant. Colchicine users demonstrate a statistically significant reduction in the risk of all-cause mortality (HR: 0.67, 95% CI: 0.54–0.84; *p* < 0.0001) and ischemic stroke (HR: 0.45, 95% CI: 0.29–0.67; *p* < 0.0001), but not cardiac arrest and AMI. No significant differences were observed in the risk estimates for composite outcomes, ventricular arrhythmias, or acute HF (Table 2; Fig. 1).

## Discussion

In this large, retrospective, propensity score-matched cohort study of patients with chronic pericarditis, as a primary outcome, we evaluated the association between colchicine use and the risk of AF over a 1 - year period. For secondary outcomes we evaluated the composite events, ischemic stroke, mortality, cardiac arrest, ventricular arrythmias, acute HR and AMI over a 1 - year period in colchicine user and non-users. No significant increase in the incidence of AF was observed over a 1 - year period in patients with chronic pericarditis treated with colchicine.

While most preclinical and clinical studies suggest that colchicine may have protective effects against arrhythmia, there is also conflicting evidence indicating that colchicine could potentially promote pro-arrhythmic effects [8, 9]. It is considered that colchicine may be beneficial in preventing AF in patients by reducing the atrial remodeling and atrial arrhythmogenesis [10, 11]. Colchicine binds to tubulin and inhibits microtubule polymerization, leading to an anti-inflammatory response and attenuating cardiac remodeling, which may be beneficial in preventing arrhythmogenesis [12]. Additionally, colchicine reduces the production of pro-inflammatory cytokines, such as interleukin-1β and interleukin-6, both of which are implicated in atrial remodeling [13]. On the other hand, a study by Kerfant et al. [14] showed that colchicine-induced microtubule disruption can impair calcium handling in ventricular myocytes, potentially predisposing these cells to arrhythmogenesis, also the NLRP3 (NACHT-, LRR- and pyrin domain-containing 3) inflammasome has been implicated in cardiomyocyte-mediated inflammatory signaling, which may contribute to the pathogenesis of AF [15]. Another study suggested that colchicine may exert pro-arrhythmic effects, possibly due to disruption of the microtubular network in rat ventricular myocytes [16].

Randomized clinical trials have failed to demonstrate a significant association between colchicine use and the incidence of AF. In the LoDoCo trial, which evaluated colchicine in patients with chronic coronary disease, colchicine was not associated with a reduced risk of new-onset or recurrent AF or atrial flutter (HR: 0.84, 95% CI 0.66–1.07) [17]. Similarly, in the COLCOT trial, which assessed the efficacy and safety of low-dose colchicine following MI, colchicine was not significantly associated with AF (HR: 0.93, 95% CI 0.59–1.43) [18]. The effect sizes observed in these randomized trials are broadly similar to those in our study, in which colchicine use was not significantly associated with a lower risk of AF. Additionally in COP-AF study which evaluated the effect of colchicine on perioperative AF after non-cardiac surgery [19], administration of colchicine did not show to effect the incidence of clinically important AF. While prior mechanistic studies suggest that colchicine may have a potential protective effect on AF due to its anti-inflammatory properties, randomized clinical studies lacked to show this effect. However, a meta-analysis evaluating the effect of colchicine on AF found a reduced incidence of AF in patients following cardiac surgery or pulmonary vein isolation/ablation suggesting a protective effect of colchicine on AF in certain patient groups [20]. Overall, it can be said that the current findings do not provide strong epidemiological evidence to support a protective effect of colchicine on AF risk in patients with chronic pericarditis.

Among patients with chronic or recurrent pericarditis, colchicine use was associated with a statistically significant (33%) relative reduction in 1-year all-cause mortality after PSM. When we look at the composite outcomes (including all-cause mortality, myocardial infarction, stroke, and ventricular arrhythmia), colchicine demonstrated a favorable trend however the observed effect did not reach statistical significance after PSM. These findings align with prior evidence from cardiovascular cohorts suggesting that colchicine’s anti-inflammatory properties may contribute to improved long-term cardiovascular outcomes in patients with chronic pericarditis [17, 18].

Additionally, the risk of ischemic stroke was markedly lower among colchicine users suggesting a potential cerebrovascular benefit. This finding may be explained by colchicine’s ability to modulate systemic inflammation, which is increasingly recognized as a key contributor to atherosclerotic plaque instability and thromboembolic events [21]. By inhibiting microtubule polymerization and suppressing the NLRP3 inflammasome, colchicine reduces the production of pro-inflammatory cytokines such as interleukin-1β and interleukin-6, both of which have been implicated in the pathogenesis of cerebrovascular disease [22]. These anti-inflammatory effects may contribute to plaque stabilization, reduced endothelial dysfunction, and decreased platelet aggregation, thereby lowering the risk of thromboembolic stroke.

Furthermore, in the context of chronic pericarditis, colchicine may prevent recurrent pericardial inflammation and effusion [23], which can lead to hemodynamic compromise or contribute to AF, an established risk factor for cardioembolic stroke [11]. Although our analysis did not demonstrate a statistically significant reduction in AF, the overall attenuation of inflammatory burden may still reduce stroke risk through other mechanisms, including improved vascular health and decreased systemic thrombo-inflammatory activity [24].

With respect to secondary endpoints such as acute HF, ventricular arrhythmias, AMI and cardiac arrest, our study did not observe statistically significant differences between colchicine users and non-users following PSM. In patients with HF, colchicine may be thought to reduce the events for HF potentially through its anti-inflammatory properties but our study failed to show such an effect. Similar to our findings, one clinical study with 267 subjects with chronic HF colchicine compared with placebo did not reduce the proportion of subjects achieving improvement in the New York Heart Association (NYHA) class, although CRP and IL6 were significantly reduced in the colchicine group [25]. In another clinical study, Pascual-Figal et al. demonstrated the anti-inflammatory benefits of colchicine but failed to show any advantage of colchicine over placebo in acute decompensated HF [26].

In studies investigating colchicine for ventricular arrythmias, Fronmeier et al. demonstrated a significant shortening of ventricular refractory period with colchicine use, which may be associated with a pro-arrhythmogenic effect of the drug [9]. In contrast, several studies have demonstrated the anti-arrhythmic effects of colchicine, supporting its potential benefit in the prevention of arrhythmias [27]. These findings suggest that colchicine does not have a significant influence on arrhythmia risk, indicating neither a protective nor a harmful effect.

Given its anti-inflammatory and plaque-stabilizing properties, colchicine may be hypothesized to reduce the incidence of AMI [28]. In LoDoCo trial colchicine found to decrease the AMI risk by 30% (HR: 0.70, 95%CI 0.53–0.93), however the COLCOT trial failed to show such an effect (HR: 0.91, 95%CI 0.68–1.21) [17, 18]. In a meta-analysis evaluating the effect of colchicine on major cardiovascular outcomes in more than 30,000 patients, colchicine use was associated with a reduced relative risk of AMI [29]. For cardiac arrest, the COLCOT trial [18], did not find any statistical difference between colchicine and placebo (HR: 0.83, 95% CI 0.25–2.73), which is broadly similar to our findings.

## Limitations

This study has several limitations that should be considered when interpreting the findings. First, although the median follow-up time was close to one year, it remained slightly below the full 12-month period, both before and after PSM. This may limit the ability to fully capture late-onset events and reduce the statistical power to detect differences in 1-year outcomes, particularly for relatively infrequent endpoints such as cardiac arrest, AMI or ventricular arrhythmia. It is also challenging to interpret certain quantitative variables such as body mass index, glomerular filtration rate, and ejection fraction, as these parameters are often inconsistently recorded or unavailable. Therefore, PSM was performed based on the presence of related diagnoses (e.g., chronic kidney disease, HF and obesity), rather than these continuous measures. In addition, smoking status is frequently missing or incompletely coded within the TriNetX system; thus, matching for smoking could not be performed. Detailed information on the dose, frequency, and duration of colchicine therapy was not available within the TriNetX platform. Since outcomes may vary based on treatment intensity or cumulative exposure, the absence of dosage data precludes any dose–response analysis and introduces uncertainty regarding the consistency and adequacy of colchicine therapy across the cohort. Also we couldn’t have detailed information on polypharmacy or concurrent medication use within the TriNetX platform, which may have influenced arrhythmia outcomes. Drug adherence cannot be assessed. As prescription records do not confirm actual medication intake, variability in patient adherence to colchicine may have diluted the observed effects and contributed to misclassification bias. Patients recorded as colchicine users may have had suboptimal compliance, thereby potentially underestimating the true effect of the drug. This study utilized hospitalized patients with chronic pericarditis as the primary cohort, a design choice aimed at reducing heterogeneity and improving diagnostic accuracy. However, this may also limit the generalizability of our findings to broader outpatient populations or individuals using colchicine for other inflammatory or cardiovascular conditions. The reliance on ICD-10-CM diagnostic codes for both exposure and outcome identification introduce the risk of misclassification bias. Diagnostic coding errors, underreporting, or variability in coding practices across institutions may affect the accuracy of patient classification and outcome ascertainment. AF events were identified through diagnostic codes available in the TriNetX database, without access to detailed clinical records or monitoring modalities. As AF can be paroxysmal and frequently asymptomatic, reliance on coded diagnoses may lead to under- or over-ascertainment of events. We were unable to distinguish AF cases detected by different methods (e.g., symptom-driven ECG, continuous device monitoring, or incidental findings), which could contribute to the relatively high event rates observed. As with all retrospective observational studies, causal inferences cannot be definitively established. The associations observed may reflect correlation rather than causation, and unmeasured variables may have influenced both colchicine prescribing and clinical outcomes.

## Conclusion

In this large, retrospective PSM analysis of patients with chronic pericarditis, colchicine use was numerically but not significantly associated with a reduced risk of AF; however, colchicine use was linked to a markedly lower risk of all-cause mortality and ischemic stroke. Whilst it appears that colchicine may not influence arrhythmic risk in this population, its anti-inflammatory properties could potentially provide important survival and cerebrovascular benefits. Further large-scale randomized clinical trials on the effect of colchicine on AF are warranted to corroborate these findings.

## Supplementary Information

Below is the link to the electronic supplementary material.

Supplementary Material 1

Supplementary Material 2

## Data Availability

No datasets were generated or analysed during the current study.
