# Supplementary figures and images for: Evaluating the risk of atrial fibrillation in patients with chronic recurrent pericarditis prescribed colchicine: Observations using TriNetX global federated research network
Source: Eur J Clin Pharmacol. 2025 Dec 18;82(1):4. doi: 10.1007/s00228-025-03925-4 (PMC12711922; doi:10.1007/s00228-025-03925-4)

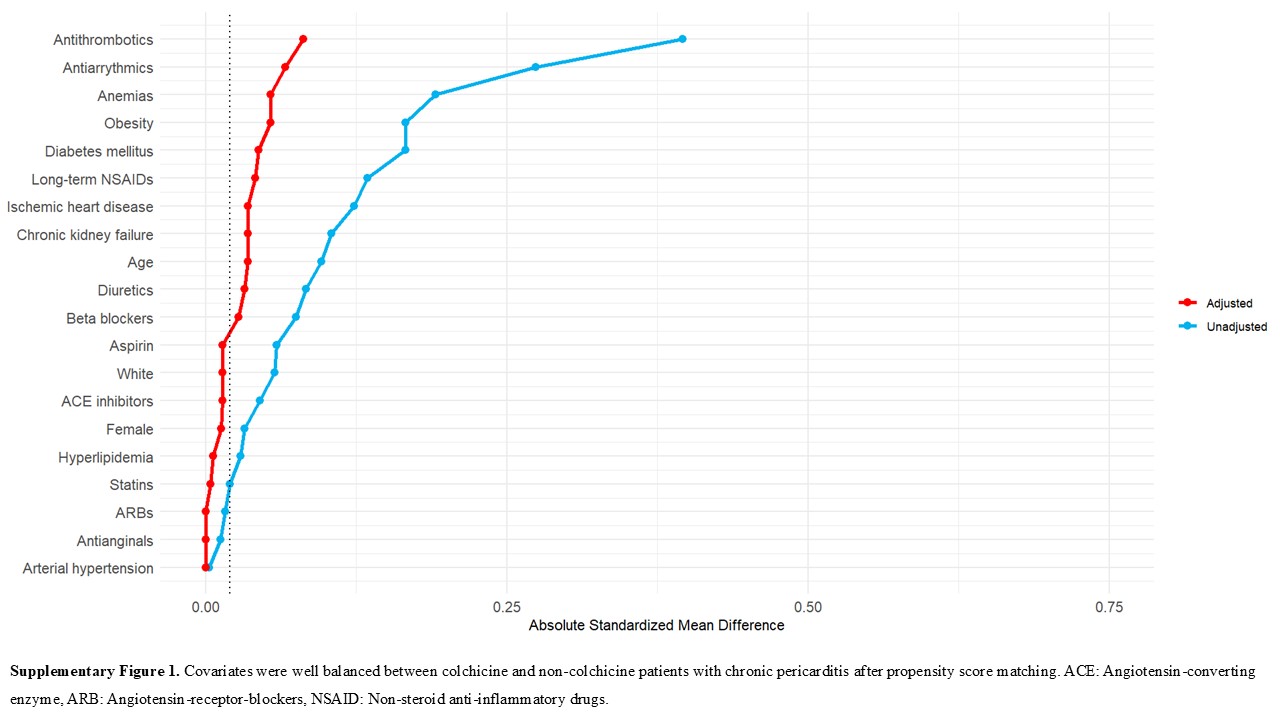

Supplement: Supplementary file 1 — Supplementary Material 1 [file 228_2025_3925_MOESM1_ESM.jpg]
