# Supplementary material for: Evaluating the risk of atrial fibrillation in patients with chronic recurrent pericarditis prescribed colchicine: Observations using TriNetX global federated research network
Source: Eur J Clin Pharmacol. 2025 Dec 18;82(1):4. doi: 10.1007/s00228-025-03925-4 (PMC12711922; doi:10.1007/s00228-025-03925-4)
Supplement: Supplementary file 2 — Supplementary Material 2 [file 228_2025_3925_MOESM2_ESM.docx]

| **Supplementary Table 1.** ICD-10-CM and ATC codes for inclusion and exclusion criteria for the study cohorts. | |
| --- | --- |
| **Patients with chronic pericarditis and colchicine use** | |
| Inclusion Criteria | 1. Hospital inpatients and observation care services between 1st January 2010 to 1st January 2024 2. Chronic adhesive pericarditis (ICD-10-CM I31.0) 3. Chronic constrictive pericarditis (ICD-10-CM I31.1) |
| After hospitalization for chronic pericarditis | |
| Inclusion Criteria | 1. Colchicine use |
| **Patients with chronic pericarditis and no-colchicine use** | |
| Inclusion Criteria | 1. Hospital inpatients and observation care services between 1st January 2010 to 1st January 2024 2. Chronic adhesive pericarditis (ICD-10-CM I31.0) 3. Chronic constrictive pericarditis (ICD-10-CM I31.1) |
| After hospitalization for chronic pericarditis | |
| Exclusion Criteria | 1. Colchicine use |
